# A Case of Persistent Hiccups Induced by an Enlarging Hepatic Cyst Underneath the Diaphragm

**DOI:** 10.7759/cureus.58278

**Published:** 2024-04-14

**Authors:** Wataru Ando, Yuki Otsuka, Fumio Otsuka

**Affiliations:** 1 Department of General Medicine, Okayama University Graduate School of Medicine, Dentistry and Pharmaceutical Sciences, Okayama, JPN; 2 Center for Graduate Medical Education, Okayama University Hospital, Okayama, JPN

**Keywords:** prolonged hiccups, liver cysts, autosomal-dominant polycystic kidney disease, hepatic cysts, hiccups

## Abstract

Although hiccups are regularly self-limited and rarely life-threatening, prolonged hiccups are bothersome, which can significantly decrease the quality of life. Here, we report a case of persistent hiccups coexisting with an enlarging liver cyst situated just below the diaphragm in a patient with autosomal dominant polycystic kidney disease (ADPKD). No other underlying etiologies related to the symptoms were suspected. The cyst was difficult to drain surgically and the patient continued with symptomatic treatment. Although rare, hepatic cysts should be considered a potential cause of prolonged hiccups in patients with ADPKD. We emphasize the significance of systematically excluding potential etiologies that cause prolonged hiccups and considering appropriate therapeutic interventions.

## Introduction

A hiccup is a quick, involuntary, and spasmodic contraction of the diaphragm and intercostal muscles that leads to inspiration and loud reflexive closure of the vocal cords [1,2]. Hiccups are usually self-limiting and temporary but can sometimes last for more than 48 h, which is called prolonged hiccups [1]. Prolonged hiccups lasting more than 48 h to one month are defined as “persistent” hiccups and hiccups lasting more than one month are called “intractable” hiccups [2]. Patients with persistent or intractable hiccups generally require an evaluation of the underlying etiologies.

Most causes of prolonged hiccups are structural abnormalities such as central nervous system (CNS) disorders, vagus or phrenic nerve irritation, and gastrointestinal, thoracic, and cardiovascular disorders [2]. Additionally, toxic metabolic, postoperative, and psychogenic factors are possible causes of hiccups [3,4]. However, few studies have reported hepatic lesions as a cause of persistent hiccups. Here, we report the case of a 66-year-old patient with an enlarged hepatic cyst, which was thought to be a potential cause of persistent hiccups.

## Case presentation

A 66-year-old man presented to a primary care physician with persistent hiccups, which persisted for a couple of days. He had end-stage renal disease complicated by autosomal dominant polycystic kidney disease (ADPKD) and had undergone hemodialysis five years ago. He was listed for kidney transplantation and regularly visited urological services.

The hiccups persisted throughout the day and prevented the patient from eating food, resulting in a weight loss of 4 kg. There were no obvious provocative or palliative factors related to the symptoms. He was initially treated with shakuyakukanzoto, a Japanese herbal medicine, which was ineffective, and then he was prescribed clonazepam. However, neither of them could stop the hiccups. Additional treatment with metoclopramide and hydroxyzine pamoate was ineffective; therefore, gabapentin was prescribed, which only slightly improved the patient’s symptoms. The patient was then referred to our department for further investigation and management.

**Figure 1:**
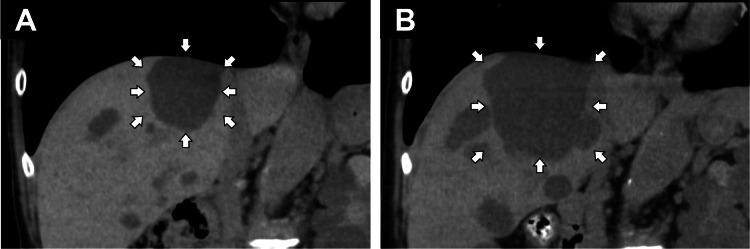
Abdominal computed tomography of hepatic cysts The cyst in the upper liver, just below the diaphragm, enlarged from 35×37 mm (A) to 63×70 mm (B) over five years.

His vital signs were as follows: body temperature of 36.5℃, a heart rate of 100 bpm, and blood pressure at 200/132 mmHg. Physical examinations of the neck, chest, and abdomen revealed no remarkable abnormalities. Laboratory examinations, including serum sodium and potassium levels, did not reveal the cause of the symptoms (Table 1). However, abdominal computed tomography revealed that one of the liver cysts, located just below the right diaphragm, was enlarged (Figure 1). A cyst in the upper liver is suspected to compress the diaphragmatic muscle and stimulate the phrenic nerve, causing hiccups. After consultation with gastroenterologists, percutaneous drainage of the cysts was considered technically difficult. The patient continued to undergo symptomatic treatment.

**Table 1:** Laboratory fndings ALP: alkaline phosphatase, ALT: alanine aminotransferase, AST: aspartate aminotransferase, Alb: albumin, Bas: basophil, CI: chloride, CRP: C-reactive protein, Ca: calcium, Cre: creatinine, Eos: eosinophil, Hb: hemoglobin, K: potassium, LD: lactate dehydrogenase, Lym: lymphocyte, MCHC: mean corpuscular hemoglobin concentration, MCV: mean corpuscular volume, Mon: monocyte, Na: sodium, Neu: neutrophil, Plt: platelet, RBC: red blood cell, T. Bil: total bilirubin, TP: total protein, UA: uric acid, UN: urea nitrogen, WBC: white blood cell

Parameters	Patient value	Reference range
WBC (/μL)	4450	3330-8600
Lym (%)	23.8	16.5-49.5
Neu (%)	62.1	40.0-70.0
Mon (%)	8.0	2.0-10.0
Bas (%)	1.4	0.0-2.5
RBC (10^6^/μL)	4.36	4.35-5.55
Hb (g/dL)	13.5	13.7-16.8
MCV (fL)	98.8	83.6-98.2
MCHC (g/dL)	31.4	31.7-35.3
Plt (10^3^/μL)	285	158-348
TP (g/dL)	7.5	6.6-8.1
Alb (g/dL)	4.2	4.1-5.1
AST (U/L)	9	13-30
ALT (U/L)	11	10-42
ALP (U/L)	77	38-113
LD (U/L)	164	124-222
T. Bil (mg/dL)	0.47	0.4-1.5
UN (mg/dL)	36.6	8.0-20
Cre (mg/dL)	10.58	0.65-1.07
UA (mg/dL)	5.1	3.9-6.9
Na (mmol/L)	139	138-145
K (mmol/L)	4.7	3.6-4.8
Cl mmol/L	102	101-108
Ca (mg/dL)	9.7	8.8-10.1
CRP (mg/dL)	0.09	<0.14

## Discussion

Hiccups, also called singultus, are symptoms that can be observed even in healthy individuals with no medical history. Many causes have been reported, including CNS disorders (vascular disease, tumors, inflammation, and trauma), vagus or phrenic nerve irritation, and gastrointestinal, thoracic, and cardiovascular disorders [2,3,5]. Medications including benzodiazepines, opiates, and dopamine agonists also induce hiccups [4,6]. Liver diseases usually do not cause hiccups, and most liver cysts are asymptomatic; only a limited number of cases of persistent hiccups due to liver abscesses have been reported [7,8].

Prolonged hiccups are usually not life-threatening; however, they are sometimes bothersome, leading to exhaustion, fatigue, malnutrition, weight loss, and dehydration [9]. To manage this, clinicians should explore the underlying etiologies because specific appropriate therapies can sometimes be administered if the etiologies are detected. The radial treatment of giant hepatic cysts involves surgical drainage. However, in some cases symptomatic treatment is the only option, as in this case. Symptomatic treatments include physical maneuvers and pharmacotherapy [2]. Physical maneuvers including breath holding, Valsalva maneuver, pulling on the tongue, biting into a lemon, and drinking water are easy to perform and rarely cause complications [10].

Baclofen and gabapentin are considered the first-line medical therapies [11]. Other anticonvulsants, antidepressants, CNS stimulants, and antiarrhythmics are also used to treat prolonged hiccups [2]. If a single dose is ineffective, a combination of these medications is considered useful. In our case, shakuyakukanzoto, a traditional Japanese herbal medicine primarily focused on alleviating muscle cramps and spasms, was administered; however, its evidence for the treatment of hiccups is limited [12].

Patients with ADPKD often have hepatic cysts as a complication but are usually asymptomatic [13]. However, this case highlights the need to focus on expanding hepatic cysts because of the potential cause of persistent hiccups. Persistent hiccups related to liver cysts are not life-threatening; however, they decrease the quality of life [14]. When patients with ADPKD complain of hiccups, physicians should rule out underlying etiologies other than hepatic cysts using laboratory and imaging examinations and consider appropriate therapeutic interventions.

## Conclusions

In summary, here we report that enlarged hepatic cysts may be responsible for prolonged hiccups. Persistent hiccups are bothersome and decrease the quality of life of the affected person, and also, they sometimes could be fatal. We emphasize the significance of systematically exploring underlying etiologies that can cause prolonged hiccups and excluding life-threatening ones.
